# The Detrimental Effects of Crystalline Excipients: How They Jeopardize the Long-Term Stability of Freeze-Dried Polypeptide Formulations

**DOI:** 10.3390/pharmaceutics17121543

**Published:** 2025-11-29

**Authors:** Han Gao, Jun Ouyang, Zhi-Bo Hu, Wei-Jie Fang

**Affiliations:** 1 Institute of Drug Metabolism and Pharmaceutical Analysis, College of Pharmaceutical Sciences, Zhejiang University, Hangzhou 310058, China; 22019065@zju.edu.cn (H.G.); 22319166@zju.edu.cn (Z.-B.H.); 2 Taizhou Institute of Zhejiang University, Taizhou 318000, China; 3 National Medical Products Administration Key Laboratory for Core Technology of Generic Drug Evaluation, Zhejiang Institute for Food and Drug Control, Hangzhou 310052, China; 22019086@zju.edu.cn; 4 Innovation Center of Translational Pharmacy, Jinhua Institute of Zhejiang University, Jinhua 321016, China

**Keywords:** amorphous excipient, crystalline excipient, freeze-drying, polypeptides, stability

## Abstract

**Background**: Despite the growing importance of polypeptide-based drugs in clinical therapy, studies investigating the stability of their freeze-dried formulations remain scarce. Crystalline excipients, such as mannitol, are commonly used in freeze-dried formulations of chemically synthesized drugs, but they often negatively impact the long-term stability of biological macromolecules like monoclonal antibodies (mAbs). This study bridges this knowledge gap by evaluating the effects of crystalline and amorphous excipients, surfactants, and amino acid-based stabilizers on the long-term stability of freeze-dried formulations using model polypeptides, glucagon and insulin. **Methods**: The freeze-dried formulations were prepared with crystalline and amorphous excipients, surfactants, and amino acid-based stabilizers. The crystallization behavior of the excipients and the thermal stability of the formulations were thoroughly characterized using X-ray powder diffraction (XRPD) and differential scanning calorimetry (DSC). **Results**: The crystallization of mannitol was directly correlated with a significant reduction in the long-term stability of both model polypeptides. This detrimental effect mirrors the instability observed in mAbs formulations, indicating a common mechanism of protein destabilization induced by crystalline excipients, independent of molecular size. **Conclusions**: This study provides the first direct evidence that crystalline excipients pose a significant risk to the stability of freeze-dried polypeptides. These findings offer critical insights for the rational design of stable freeze-dried formulations, guiding industrial development strategies for polypeptide-based therapeutics.

## 1. Introduction

In recent decades, advances in synthesis and delivery technologies for polypeptide-based drugs have significantly promoted their development as promising therapeutic agents. Notable examples include insulin, semaglutide, and tirzepatide [1,2,3,4]. Semaglutide, in particular—approved for glucose lowering and weight management—ranked among the top-ten best-selling drugs worldwide by revenue in 2024 [5,6,7]. However, due to their intermediate molecular size, which falls between that of conventional small-molecule drugs (SMDs) and monoclonal antibodies (mAbs), therapeutic polypeptides often exhibit inferior stability compared to SMDs. Conventional perspectives frequently assumed that polypeptides possess stability comparable to that of SMD, thereby overlooking instability mechanisms shared with proteins and mAbs, such as susceptibility to aggregation and degradation. Like mAbs, polypeptides drugs are vulnerable to various external stressors during manufacturing, transportation, and storage, which can induce both chemical and physical degradation [8,9,10,11]. Chemical instability in polypeptides typically involves covalent modification, such as oxidation, deamidation or polypeptide backbone hydrolysis [12]. In contrast, physical instability, a hallmark of biopharmaceuticals, primarily arises from aggregation, unfolding, adsorption, precipitation, or fragmentation [12,13]. Owing to low oral bioavailability, most polypeptide and protein therapeutics require repeated administration via injection, typically formulated as solutions or suspensions. However, polypeptides in solution exhibit limited shelf life. Moreover, physical aggregation can lead to elevated levels of subvisible particles, raising potential safety concerns for injection [14]. The stability profile of each therapeutic polypeptide is uniquely determined by its amino acid sequence. For instance, residue such as cysteine, methionine, or tryptophan are susceptible to oxidation, a process accelerated by freeze–thaw cycles and elevated pH conditions, ultimately compromising therapeutic efficacy [15]. Additionally, aspartic acid residues are prone to hydrolysis, while aromatic amino acids such as phenylalanine and tryptophan undergo photochemical degradation [16]. Even minor alterations in the unique amino acid sequence and chemical structure of a polypeptide can potentially lead to a complete loss of its therapeutic function.

Freeze-drying (FD) has traditionally been employed to improve the long-term storage stability of unstable therapeutic high-molecular-weight biopharmaceuticals, particularly protein drugs, by mitigating their inherent instability [17,18,19]. FD process commonly consists of freezing, primary drying, and secondary drying, with an optional annealing step if necessary. Throughout the FD process, the removal of water molecules disrupts the original hydrogen bonds between proteins and water, which may lead to protein inactivation unless appropriate formulation strategies are adopted. Therefore, incorporating excipients (such as disaccharides, amino acids, buffers, and surfactants) into protein formulations is considered a critical strategy in FD design to mitigate interfacial stresses and maintain protein stability during freezing, drying, and long-term storage [20,21,22]. Traditionally, nonreducing disaccharides such as trehalose and sucrose have been widely used as an essential stabilizer in freeze-dried protein formulations. Studies have concluded that these sugars can effectively inhibit the protein unfolding and maintain the native structure of proteins during the freezing and drying. More importantly, they form a stable glassy matrix in the solid state, which is critical for extending the shelf life of lyophilized products. On the other hand, crystalline excipients such as mannitol are commonly incorporated as bulking agents in freeze-dried formulations of both SMD and mAbs. Owing to its favorable physicochemical characteristics, mannitol is utilized in various solid dosage forms. Key applications include: (i) serving as a diluent in tablet formulations, owing to its non-hygroscopic property and brittle fracture behavior under compression, and (ii) incorporation into chewable tablets due to its cooling effect, sweet taste, and pleasant mouthfeel [23,24,25,26,27]. Yet during the freezing step of the FD process, solute concentration can induce the crystallization of mannitol, potentially disrupting the native structure of proteins at the ice–water interface. Moreover, the crystalline forms generated—particularly mannitol hemihydrate (MHH)—have been reported to adversely affect the long-term stability of mAbs. Multiple studies indicate that the presence of MHH in the lyophilized cake can compromise the stability of thermolabile therapeutic components [28,29,30,31].

The stabilizing and destabilizing capacities of various excipients in different high-molecule-weight protein formulations and the mechanism for such stabilization were reported by extensive literature [32,33,34,35,36]. In the field of freeze-dried mAb formulations, crystalline excipients such as mannitol are conventionally utilized as bulking agents. However, their crystallization tendency may disrupt the native structure of mAbs at the ice-water interface and fail to provide a protective amorphous matrix during long-term storage. In contrast, amorphous stabilizers like sucrose are preferentially employed in freeze-dried mAb formulations, where they form a stable amorphous matrix that effectively mitigates protein–protein interactions, thereby ensuring enhanced long-term stability. Nevertheless, there is limited literature describing the excipients effects on the stability of polypeptide drugs in the freeze-dried formulations [8,37,38,39,40]. In one study, Santana et al. reported that the degradation rate of in recombinant human epidermal growth factor assessed by reversed-phase-high performance liquid chromatography could decrease 100 times at 37 °C and 70 times at 50 °C in freeze-dried with respect to aqueous formulation [41]. And an increase in freeze-dried recombinant human epidermal growth factor stability was observed with the increase in protein concentration from 25 to 250 μg/vial, which demonstrating the polypeptide stability may correlated with the concentration [42]. In addition, through a screening of combinations comprising amorphous excipients and surfactants, Fang et al. concluded that polypeptides may exhibit distinct behaviors compared to proteins due to their smaller molecular size and less ordered secondary structure [8]. Although a limited number of studies have investigated freeze-dried polypeptides, a systematic comparison of the stabilization and destabilization mechanisms induced by crystalline excipients in polypeptide versus monoclonal antibody formulations remains lacking. To address this research gap, this study aims to systematically investigate the long-term stability of freeze-dried polypeptides.

In this study, insulin and glucagon were selected as model polypeptide for FD and long-term accelerated stability experiments. These hormones represent foundational therapeutics in their respective clinical areas and rank among the most commercially successful polypeptide-based drugs to date. Insulin, a 51-amino acid (AA) polypeptide, plays a critical role in maintaining blood glucose homeostasis and serves as an essential therapy for both type I and II diabetes [43,44]. Glucagon, a 29-AA polypeptide secreted by pancreatic α-cells in response to hypoglycemia, elevates blood glucose levels by promoting hepatic gluconeogenesis and glycogenolysis [45,46].

The present work systematically investigates the effects of key excipients (such as crystalline/amorphous stabilizers, surfactants, and arginine) on the stability of freeze-dried formulations using insulin and glucagon as model systems. Our central hypothesis posits that the stabilization mechanisms conferred by excipients differ substantially between low-molecular-weight polypeptides and high-molecular-weight mAbs, owing to inherent differences in molecular size and structural complexity. The polypeptide aggregation was monitored using a complementary set of techniques, including size exclusion-high performance liquid chromatography (SE-HPLC), dynamic light scattering (DLS), and micro-flow imaging (MFI). Concurrently, polypeptide degradation was assessed by reversed-phase-high performance liquid chromatography (RP-HPLC). Through a systematic, cross-scale comparison, this study aims to elucidate these distinct interaction mechanisms. The findings are anticipated to establish a critical theoretical foundation for rational formulation design principles specifically tailored to polypeptide-based therapeutics.

## 2. Materials and Methods

### 2.1. Materials

Glucagon (purity: 98%) was purchased from Science Polypeptide Biological Technology Co., Ltd. (Shanghai, China). Insulin (purity: 98%) was purchased from Hisun Pharmaceutical Co., Ltd. (Taizhou, China). Mannitol was obtained from Aladdin Industrial Corporation (Shanghai, China). Sodium chloride, and hydrochloric acid were purchased from Sinopharm Chemical Reagent (Shanghai, China). Trehalose was obtained from Sinozyme Biological Technology Co., Ltd. (Nanjing, China). Arginine hydrochloride was obtained from GPC Biological Technology Co., Ltd. (Beijing, China). HES was obtained from Macklin Biochemical Co., Ltd. (Shanghai, China). Sorbitol was obtained from Alpha Hi-tech Pharmaceutical Co., Ltd. (Jiangxi, China). Polysorbate 20 was obtained from JTBaker (Lardner, IL, USA). We purchased 0.22 μm filter membranes from Millipore Co., Ltd. (Nantong, China). Gray butyl stoppers (13 mm) and borosilicate type I scintillation vials (2 mL) were purchased from West Pharmaceuticals (Singapore) and Schott (Suzhou, China), respectively.

### 2.2. Methods

#### 2.2.1. Formulations Preparation

Glucagon (2 mg/mL) was weighted and dissolved in sodium citrate buffers, 5 mM, pH 3.0. Insulin (2 mg/mL) was weighted and dissolved in Tris, 10 mM, pH 7.5. To reduce the adverse effects of direct dissolution of excipients on protein production, the solution was mixed with different excipients solutions (prepared the same buffer solution with twice the concentration of excipients beforehand) in a 1:1 (*v*/*v*) ratio. The formulations were listed in Table 1. The solution was filtered through 0.22 μm Millipore membrane prior to filling. All protein concentrations of samples are 1 mg/mL.

#### 2.2.2. Freeze-Drying

All glass vials were thoroughly rinsed with filtered deionized ultrapure water 3 times and dried at 120 °C for at least 12 h prior to use. All samples were added to 2 mL vials on a clean bench. The samples were freeze-dried using the VirTis Advantage freeze-dryer (SP Scientific, Los Angeles, CA, USA). A 30 min equilibration at 5 °C was performed before the start of the FD process. The overview of FD cycle parameters was provided in Table 2. The vials were stoppered at 60 mTorr. After FD cycle, all freeze-dried samples were sealed with aluminum caps and stored at 4 °C.

#### 2.2.3. Karl-Fisher Moisture Determination

The residual moisture content in the freeze-dried samples was determined using HEGONG AKF-V6 KF moisture titrator (Shanghai HEGON Scientific Instrument Co., Ltd., Shanghai, China) in a dry environment. Blank corrections were applied. All samples were detected for triple measurements. The standard deviation (SD) of repeated measurements was less than 0.2% (*w*/*w*).

#### 2.2.4. Differential Scanning Calorimetry (DSC)

The DSC5A (Waters, DE, USA) coupled with a refrigerated cooling system was used to determine the glass transition temperatures of the frozen solutions (T_g_′) and of the freeze-dried solids (T_g_). All freeze-dried samples and liquid sample prepared in 40 μL pans were weighed and hermetically sealed. For the measurement of T_g_′, the solution was cooled to −60 °C, held for 5 min and then heated to 25 °C at 10 °C/min. For the measurement of T_g_, the samples were first heated to above T_g_ to remove the thermal history, and then cooled back to 0 °C, and then rescanned at 20 °C/min to 250 °C. The thermogram obtained during the second scan was used to detect T_g_′ and T_g_ which was determined as the midpoint of the transition. All samples were detected for triple measurements.

#### 2.2.5. X-Ray Powder Diffraction (XRPD)

The XRPD measurements on freeze-dried samples were examined by Bruker D8 Advance (Bruker, Bremen, Germany). Diffractograms were obtained using a Cu-Kα radiation source (40 kV, 40 mA). Powder samples were measured in high throughput mode using a well plate reader. The diffractograms were collected in transmission mode from 5 to 40° 2θ with a step size of 0.02° (timer per step = 1 s). The reflections used to identify various forms of mannitol were based on the structure available in the Cambridge Structural Database: 13.6 and 17.3° 2θ for α-mannitol, 14.6, 16.8 and 23.4° 2θ for β-mannitol, 9.7 and 24.7° 2θ for δ-mannitol, and 9.6, 16.5, 17.9 and 25.7° 2θ for MHH [28,47,48].

#### 2.2.6. Accelerated Stability Test

The freeze-dried samples were transferred to a stability chamber maintained at 40 °C and 50 °C. After storage for preset times (1, 2, and 3 months), the samples were reconstituted and evaluated by visual inspection and chromatography analysis.

#### 2.2.7. Reverse Phase-High Performance Liquid Chromatography

The Agilent 1260 HPLC system (Agilent, Santa Clara, CA, USA) with an Elite C18 chromatography column (Dalian Elite Analytical Instruments Co., Ltd., Dalian, China) was used to qualitatively evaluate the purity of proteins. For each formulation and time point, three independent sample vials were withdrawn and analyzed separately. The elution gradient of mobile phase was listed in Table 3.

For glucagon analysis, the mobile phase comprised solvent A (water containing 10 mM ammonium formate and 0.05% formic acid) and solvent B (acetonitrile containing 0.1% formic acid). The gradient program followed the profile shown in Table 3. Other chromatographic conditions included: injection volume, 15 μL; flow rate, 0.5 mL/min; column temperature, 30 °C; detection wavelength, 220 nm; run time, 45 min.

The chromatographic separation of insulin was carried out using solvent A (water containing 15 mM ammonium formate and 0.05% formic acid) and solvent B (acetonitrile). The gradient elution was performed according to Table 3. The following parameters were applied: injection volume, 8 μL; flow rate, 0.5 mL/min; column temperature, 35 °C; detection wavelength, 214 nm; total run time, 48 min.

#### 2.2.8. Size Exclusion-High Performance Liquid Chromatography

An Agilent 1100 HPLC system (Palo Alto, CA, USA) was applied to determine the physical degradation of the glucagon and insulin. The mobile phase for insulin analysis consisted of a mixture of acetonitrile:water containing 0.1% arginine:glacial acetic acid = 20:65:15 (*v*/*v*/*v*). Before use, the mobile phase was filtered through a 0.22 μm membrane and degassed by sonication for 15 min under 350 W power. Samples were centrifuged at 8000× *g* (4 °C) for 8 min prior to injecting 10 μL of sample into a TSK-GEL G2000 WXL column (Tosoh, Tokyo, Japan). The chromatographic conditions were as follows: flow rate, 0.5 mL/min; column temperature, 25 °C; detection wavelength, 276 nm; total run time, 35 min. For each formulation and time point, three independent sample vials were withdrawn and analyzed separately.

#### 2.2.9. Dynamic Light Scattering

Particle size changes in proteins during accelerated stability studies were characterized using a Malvern Nano-ZS instrument (Malvern Panalytical, Malvern, PA, USA). For DLS measurements, 200 µL of reconstituted sample solution was loaded into a micro quartz cuvette. Prior to analysis, samples were equilibrated at 25 °C for 120 s. For each formulation, three independent samples were prepared and analyzed, with each sample measured in triplicate. Each measurement consisted of 11 consecutive scans, and the data were averaged.

#### 2.2.10. Micro-Flow Imaging

Reconstituted samples were analyzed using a 5200 MFI system (ProteinSimple, Santa Clara, CA, USA). Before each measurement, the flow cell was thoroughly rinsed with ultrapure water at maximum flow rate until the field of view was clear. Background calibration was performed prior to sample analysis. Approximately 0.6 mL of sample was transferred from the original container into the Luer inlet of the system using a 1 mL filtered pipette tip. To avoid dilution effects from residual solution, the first 0.2 mL of sample was discarded and not included in the analysis. Data were processed using the accompanying 1.1.0.24 MFI View System Suite software, which provides dynamic tool sets supporting high-throughput data analysis. Each sample was analyzed in triplicate, and the average cumulative particle counts per milliliter were calculated for the following size ranges: 2–5 µm, 5–10 µm, 10–25 µm, and 25–300 µm.

#### 2.2.11. Fluorescence-Based Fibrillation Assay

A 50 mM Tris-HCl buffer (pH 7.4) was prepared in advance. For the assay, 90 μL of the Tris-HCl buffer, 5 μL of the reconstituted glucagon solution, and 5 μL of the diluted Thioflavin T (ThT) solution were successively added to each well of a black 96-well plate to achieve a final ThT concentration of 10 μM. The plate was protected from light and incubated for 3 min. For each formulation at each accelerated stability time point, the measurement was performed in quadruplicate, with blank controls included. Fluorescence intensity was measured using a microplate reader with an excitation wavelength of 440 nm and an emission wavelength of 482 nm. The data were recorded and analyzed graphically.

## 3. Results and Discussions

### 3.1. Cake Appearance by Visual Inspection

**Figure 1:**
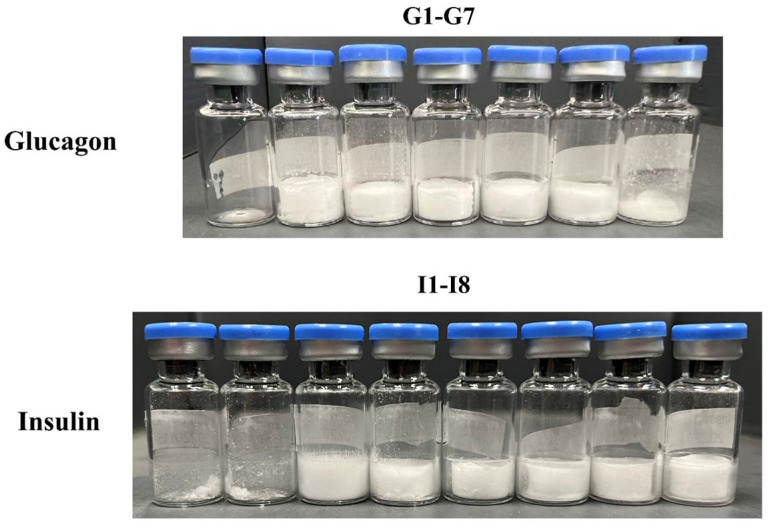
The cake appearance of different freeze-dried samples.

The cake appearance was visually inspected and evaluated by photographing through the glass vial (Figure 1). A well-formed and structurally intact freeze-dried cake generally indicates appropriate FD parameters and a stable process [49,50]. For all polypeptides in this FD cycle, as expected, the samples containing no excipients or only 0.02% (*w*/*v*) polysorbate 20 exhibited significant collapse after FD due to low T_g_′. In contrast, all 40 mg/mL trehalose-based formulations, even when supplemented with small amounts of polyols or arginine, exhibited intact cake appearance after FD. Similarly, formulation (G4, I5) containing 40 mg/mL hydroxyethyl starch (HES) also showed elegant cake appearance. Although slight shrinkage was observed in formulations prepared with amorphous excipients (trehalose and HES), the overall structural integrity of the cakes was maintained, which is considered acceptable in freeze-dried products. In addition, due to the low T_g_′ of arginine, the corresponding freeze-dried formulations (G7) also underwent noticeable collapse during primary drying. Conversely, formulations incorporating mannitol as a traditional bulking agent (G2, I3) produced most intact and elegant cakes. Notably, the influence of excipient composition on the appearance of the freeze-dried cakes was consistent across the two different samples studied.

### 3.2. The Residual Moisture of Freeze-Dried Samples

Low residual moisture is critical for ensuring the long-term stability of freeze-dried biopharmaceuticals [51]. As summarized in Table 4, formulations containing excipients exhibited significantly lower residual moisture compared to the excipient-free controls (G1 and I1). The addition of polysorbate 20 alone (I2) did not contribute to residual moisture reduction in the freeze-dried powders. This can be attributed to the absence of any structural excipients or bulking agents in these formulations, resulting in an excessively low overall T_g_′. As a consequence, the frozen matrix collapsed during primary drying, preventing the formation of a porous cake structure. The absence of open channels hindered efficient water vapor escape during sublimation, ultimately leading to both structural collapse and elevated residual moisture. Although arginine-containing formulations (G7) also underwent collapse due to their low T_g_′, their residual moisture remained within an acceptable range (below 3.0%), albeit slightly higher than those of other formulations. All other formulations containing mannitol, trehalose, or HES as primary components showed normal residual moisture levels.

### 3.3. The Physicochemical Properties of Freeze-Dried Samples

**Figure 2:**
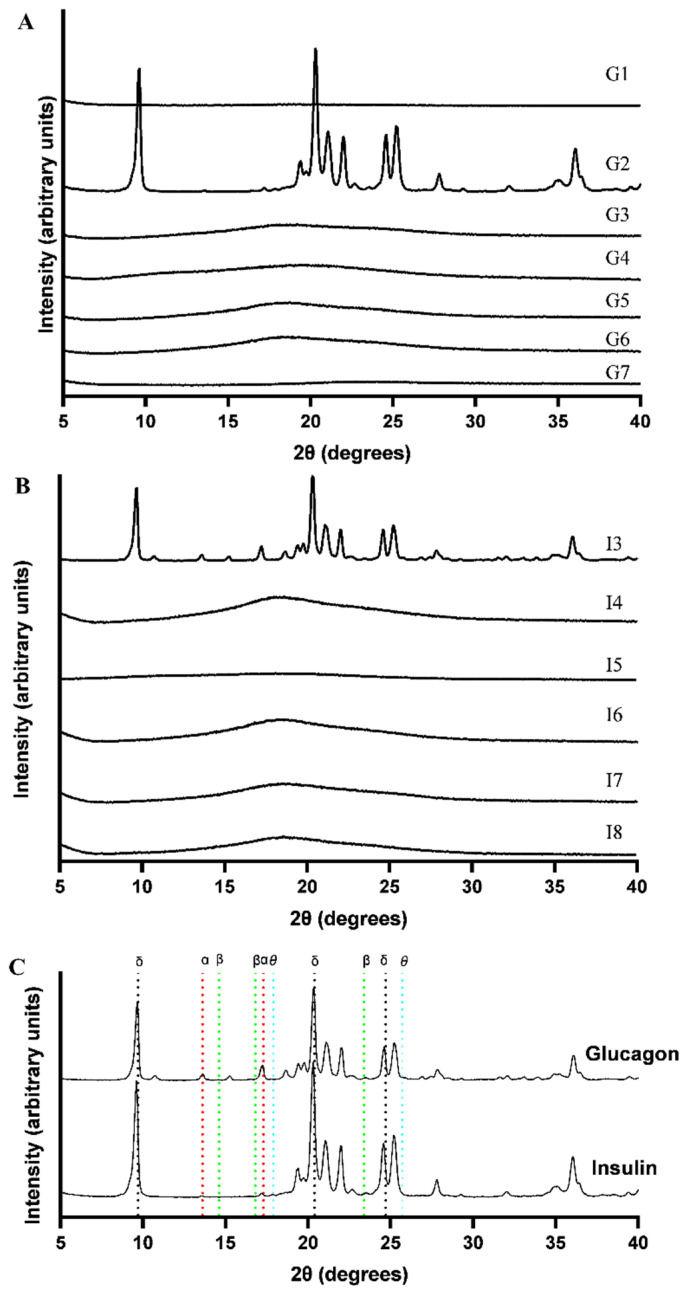
The XRPD diffractograms of freeze-dried glucagon (**A**) and insulin (**B**) proteins of different formulations. And the comparison of mannitol crystallization in G2 and T3 (**C**). Due to the collapse of the cake after FD with blank formulations and only containing polysorbate 20, and the absence of crystalline substances, XRPD were not conducted on I1, I2. α represents α-mannitol, β represents β-mannitol, and δ represents δ-mannitol, θ represents MHH. There is slight MHH signal in the freeze-dried insulin samples (I3).

To evaluate the relative crystallization tendencies of excipients, DSC was used along with XRPD. In all formulations containing 40 mg/mL mannitol (G2 and I3), crystallization of mannitol was detected by XRPD, which can be attributed to the inclusion of an annealing step in the FD process (Figure 2). In addition, slight MHH signal was observed in the freeze-dried insulin samples (I3) while no MHH signal was found in freeze-dried glucagon samples. Previous studies have confirmed that several process parameters—such as solution volume, freezing protocol, and solute concentration—were observed to influence the crystallization behavior of arginine [52]. In this study, crystallization of arginine was not detected in formulation G7. Regarding formulations containing trehalose (G3, G5 and G6), no crystallization was observed in any glucagon formulations.

The glass transition behavior of the excipients was further investigated by DSC. The T_g_ and T_g_′ are summarized in Table 5 and Table 6, respectively. Formulations containing 40 mg/mL hydroxyethyl starch (G4 and I5) exhibited the highest T_g_ values, approximately 200 °C, indicating high thermal stability in principle. In contrast, formulations with 40 mg/mL trehalose (G3 and I4) showed T_g_ values around 70 °C. Notably, the T_g_ of formulations containing only arginine was 46.9 °C. Additionally, DSC results revealed that the addition of appropriate amounts of sorbitol or arginine hydrochloride to the base formulation with 40 mg/mL trehalose led to a reduction in the overall T_g_ of the freeze-dried powder, suggesting compromised thermal stability.

### 3.4. Effect of Formulations on Long-Term Stability of Freeze-Dried Samples

**Figure 3:**
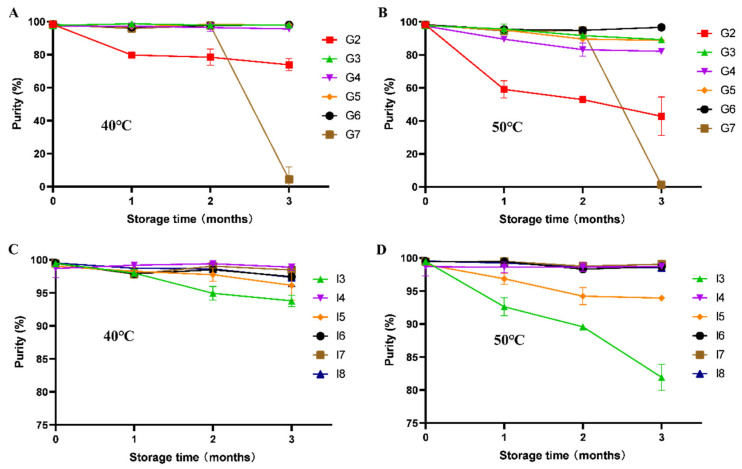
The RP-HPLC purity of polypeptides during accelerated stability study of different temperatures: glucagon (**A**,**B**), insulin (**C**,**D**). All formulations were performed in triplicate and some error bars were smaller than the symbols (n = 3).

**Figure 4:**
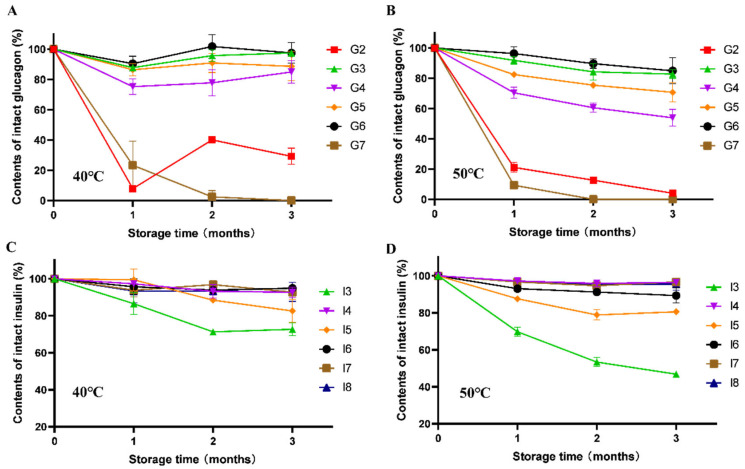
The RP-HPLC recovery of polypeptides during accelerated stability study of different temperatures: glucagon (**A**,**B**), insulin (**C**,**D**). All formulations were performed in triplicate and some error bars were smaller than the symbols (n = 3).

This study systematically investigated the purity changes in various freeze-dried formulations containing different crystalline/amorphous excipients during accelerated stability testing (Figure 3 and Figure 4).

The trehalose in insulin formulations (I4, I6, I7, and I8) was confirmed to be amorphous by XRPD, with the freeze-dried products demonstrating remarkable physical and chemical stability in long-term accelerated studies. The protective efficacy of amorphous excipients was evaluated under accelerated conditions. Under accelerated conditions, both trehalose and hydroxyethyl starch (HES) effectively maintained the purity and recovery of insulin and glucagon over three months at 40 °C. However, their stabilizing performance diverged markedly at 50 °C: trehalose-based formulations proved significantly more effective than HES-based ones, as reflected by considerably smaller reductions in polypeptide purity and recovery, highlighting its superior capacity to resist thermal stress. In contrast, the mannitol-containing formulation (I3 and G2), where RP-HPLC data showed a significant decrease in purity and recovery over storage time. The arginine-containing formulation G7, although maintaining an amorphous state, exhibited severe cake collapse at the 3-month time point during stability studies (figure not shown) due to its markedly low glass transition temperature (approximately 45 °C), which was accompanied by a sharp decline in both purity and recovery rate.

### 3.5. Effect of Formulation on Polypeptide Fibrillation

**Figure 5:**
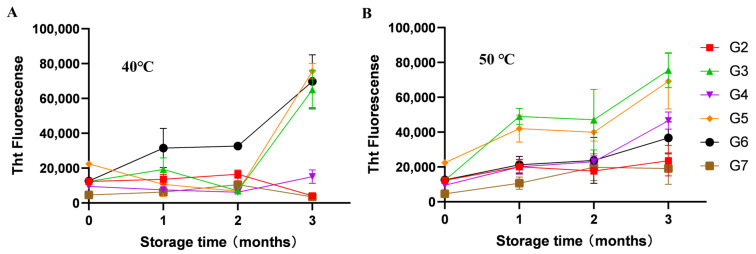
Fibrosis of freeze-dried glucagon in accelerated stability study: 40 °C (**A**) and 50 °C (**B**). All formulations were performed in triplicate and some error bars were smaller than the symbols (n = 3).

The extent of glucagon fibrillation was evaluated as a key indicator of its physical stability. As shown in Figure 5, Tht fluorescence assays revealed that G3, G5, and G6 displayed pronounced fibrillation propensity throughout the storage period. Notably, trehalose—a conventional lyoprotectant for proteins—did not provide substantial protection against fibrillation in freeze-dried glucagon. In contrast, polysorbate 20 effectively maintained glucagon’s physical stability even in the absence of other protective excipients. Throughout the accelerated stability study, the formulation containing polysorbate 20 (G1) showed no significant increase in fibrillation, suggesting a specific inhibitory effect of this surfactant on glucagon fibrillation. These findings provide new insights for optimizing the formulation stability of freeze-dried glucagon-based therapeutics.

### 3.6. Effect of Formulation on Polypeptide Aggregation

**Figure 6:**
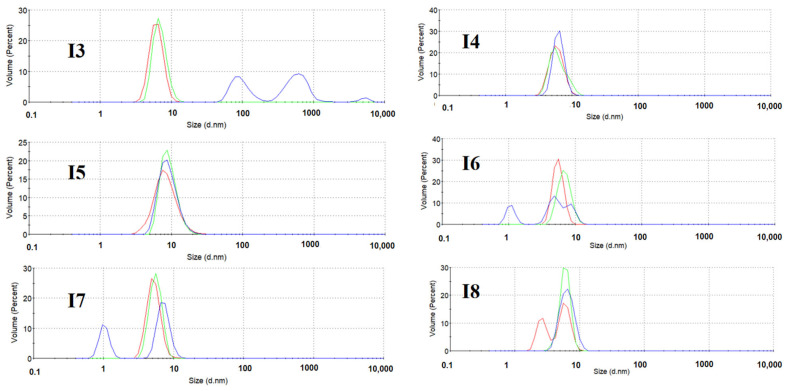
The distribution of particles detected by DLS in freeze-dried insulin with different formulations: undamaged samples (red line), samples damaged at 40 °C for 3 months (green line), and samples damaged at 50 °C for 3 months (blue line). All samples were measured in triplicate, and the average curves are shown.

SE-HPLC and DLS are well-established analytical techniques for characterizing protein aggregation behavior. As shown in Figure 6, DLS analysis revealed that the hydrodynamic diameter distribution of insulin monomers in freeze-dried formulations predominantly ranged between 6–8 nm, consistent with previously reported values [53]. During accelerated stability testing, most formulations maintained excellent stability, with particle size distributions remaining within the 6–8 nm range. However, the mannitol-containing formulation (I3) exhibited clear signs of aggregation after 3 months of storage at 50 °C, as evidenced by the emergence of two distinct peaks corresponding to larger particle sizes in the DLS profile.

**Figure 7:**
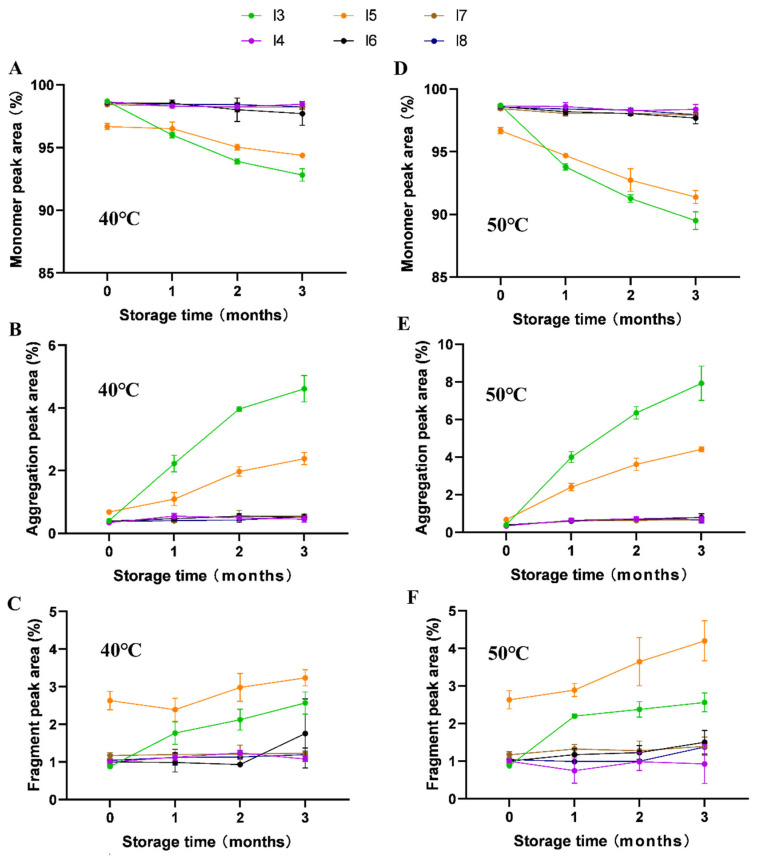
Changes in the peak area percentages of monomers (**A**), aggregates (**B**), and fragments (**C**) for freeze-dried insulin formulations with different formulations during accelerated stability testing at 40 °C, as well as changes in the peak area percentages of monomers (**D**), aggregates (**E**), and fragments (**F**) for freeze-dried insulin formulations with different formulations during accelerated stability testing at 50 °C (n = 3).

**Figure 8:**
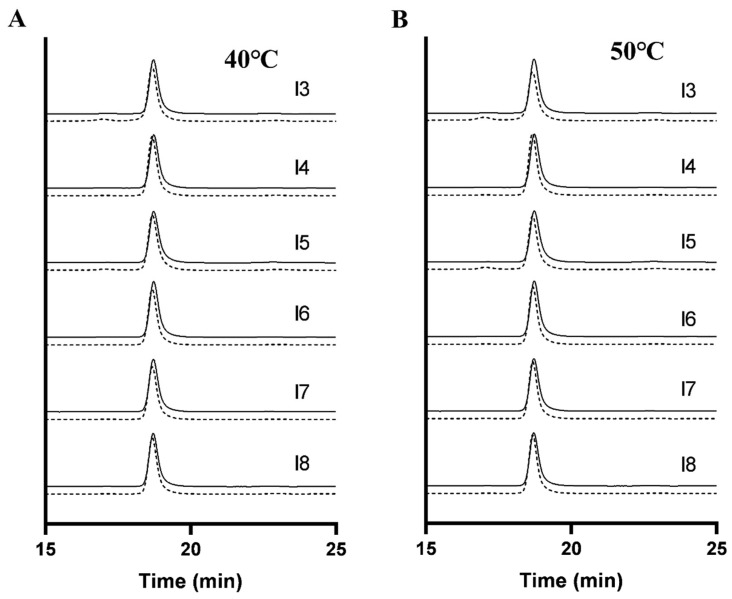
Typical SE-HPLC chromatograms of different formulation freeze-dried insulin preparations before and after degradation at 40 °C (**A**) and 50 °C (**B**): The solid line represents the undamaged sample, and the dashed line represents the sample that has undergone an accelerated stability test for 3 months.

SE-HPLC analyses (Figure 7 and Figure 8) demonstrated distinct aggregation patterns among formulations. Non-trehalose formulations (I1, I2, I3, and I5) showed a rapid increase in aggregate content under accelerated conditions. In contrast, trehalose-based formulations (I4, I6, I7, and I8) exhibited markedly slower aggregation kinetics, confirming trehalose’s role in suppressing insulin aggregation and enhancing physical stability. Notably, the HES-containing formulation (I5) displayed significantly higher fragmentation levels (initial fragment peak area: 2.63%) compared to other formulations, with further increase to 4.20% after 3 months at 50 °C (Figure 7C,F). Similarly, non-trehalose formulations (I1, I2, and I3) showed progressive fragmentation during stability testing, indicating their inability to maintain insulin’s physical stability.

A comparative analysis highlighted that SE-HPLC provided a more comprehensive assessment of physical stability changes compared to DLS. This discrepancy likely originates from fundamental distinctions in detection principles and sensitivity ranges between the two techniques. While DLS only detected aggregates in the mannitol-containing formulation (I3), SE-HPLC identified subtle fragmentation and aggregation in other formulations below DLS detection limit. These results underscore the superior sensitivity and accuracy of SE-HPLC in monitoring polypeptide aggregation and degradation.

**Figure 9:**
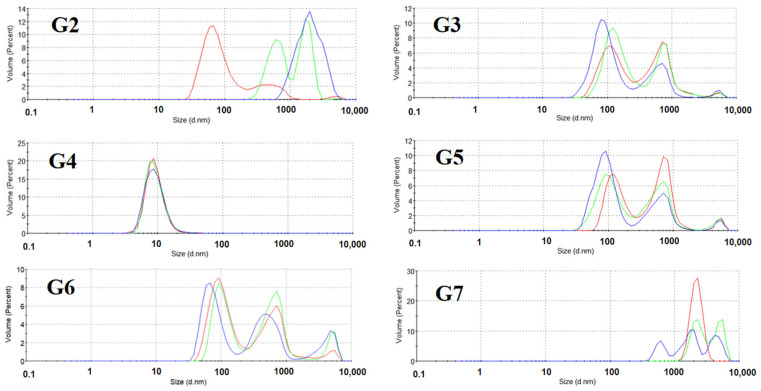
The distribution of particles detected by DLS in freeze-dried glucagon with different formulations: undamaged samples (red line), samples damaged at 40 °C for 3 months (green line), and samples damaged at 50 °C for 3 months (blue line). All samples were measured in triplicate, and the average curves are shown.

As evidenced by the DLS analysis of glucagon (Figure 9), the dominant peak observed in the range of 70–90 nm suggests that the FD process may contribute to the formation of glucagon aggregates, resulting in an increased hydrodynamic diameter. It is noteworthy that formulations containing trehalose as the primary protective agent (G3, G5, and G6) demonstrated excellent physical stability throughout the accelerated stability study, maintaining consistent nano-scale size distribution profiles. These results indicate that trehalose plays a crucial role in preserving the nanostructural stability of glucagon under the tested conditions.

### 3.7. Effect of Formulation on Subvisible Particle Formation in Polypeptide

Subvisible particles represent a critical quality attribute for injectable formulations, with significant implications for both therapeutic efficacy and patient safety. While smaller particles may compromise product potency, larger particulates (>10 μm) can potentially elicit adverse immune responses, including life-threatening anaphylaxis. Previous studies had not detected the subvisible particle in the freeze-dried polypeptides formulations. In this study, MFI was employed to systematically evaluate particle formation in glucagon and insulin formulations under accelerated stability conditions (40 °C and 50 °C for 3 months).

**Figure 10:**
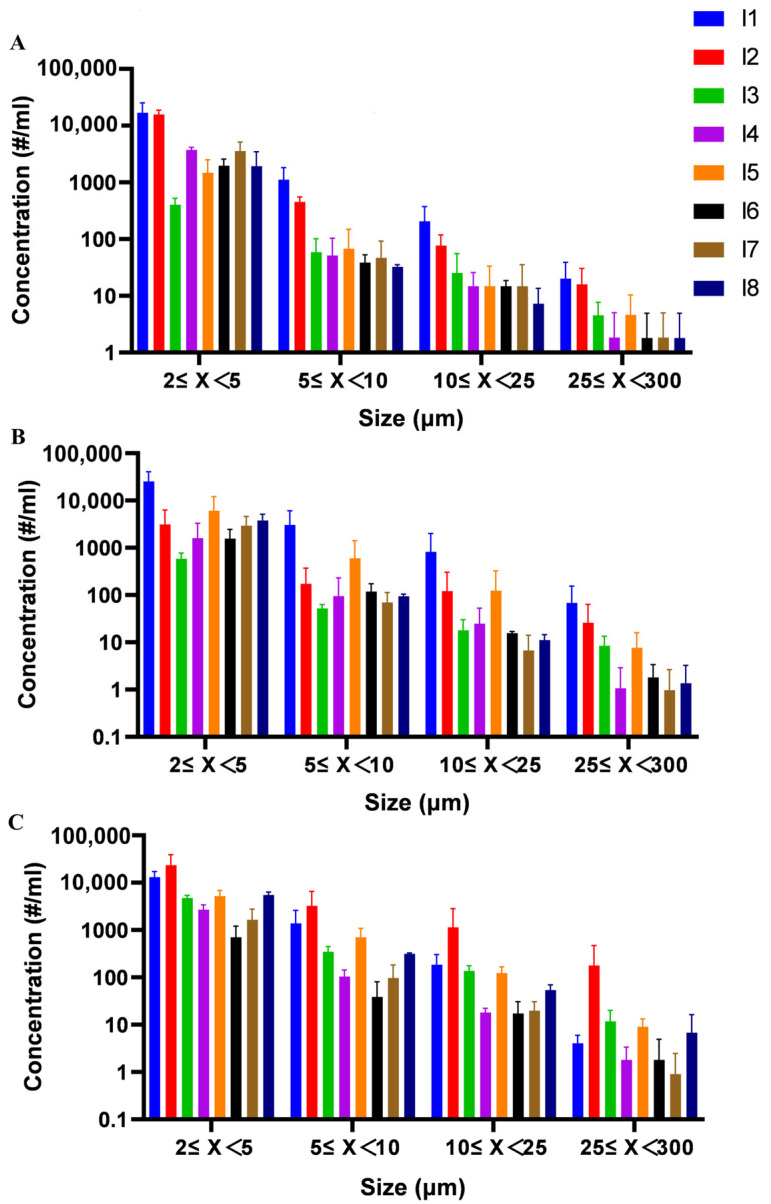
The distribution of sub-visible particles in the freeze-dried insulin of different formulations detected by MFI: undamaged samples (**A**), the sample damaged at 40 °C for 3 months (**B**) and the sample damaged at 50 °C for 3 months (**C**). # means the number of sub-visible particle. All formulations were performed in triplicate and some error bars were smaller than the symbols (n = 3).

**Figure 11:**
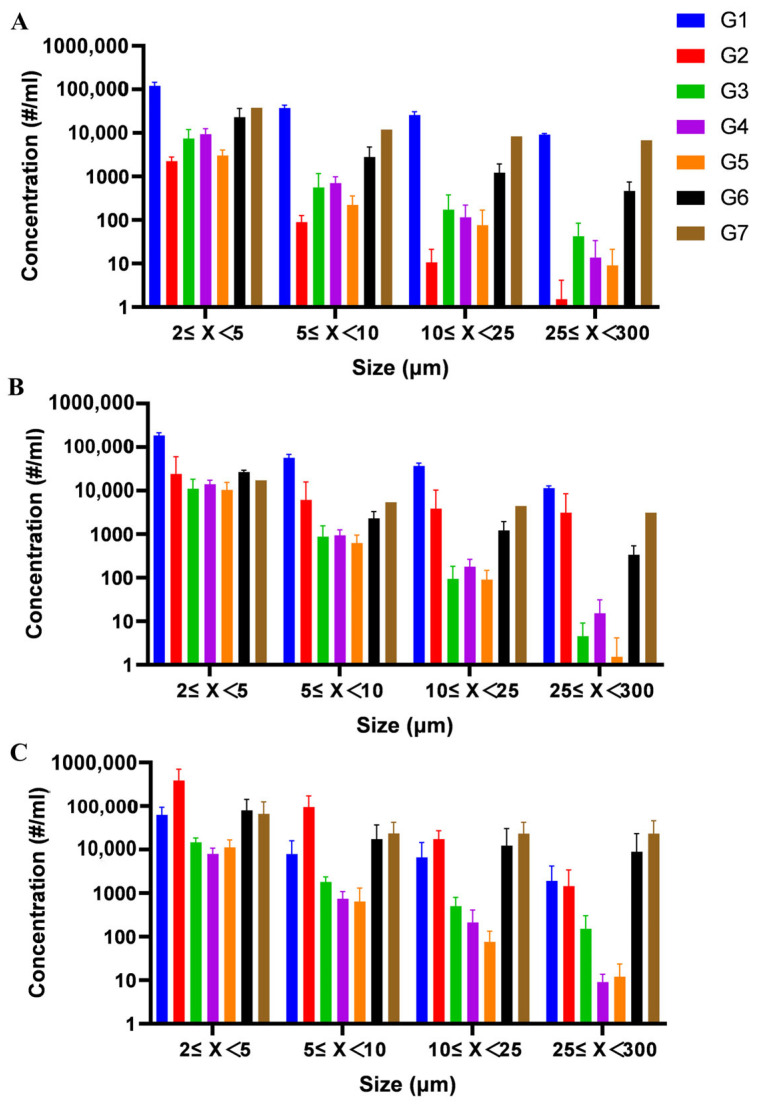
The distribution of sub-visible particles in the freeze-dried glucagon of different formulations detected by MFI: undamaged samples (**A**), the sample damaged at 40 °C for 3 months (**B**) and the sample damaged at 50 °C for 3 months (**C**). # means the number of sub-visible particle. All formulations were performed in triplicate and some error bars were smaller than the symbols (n = 3).

Trehalose-containing insulin formulations (I4, I6, I7, I8) demonstrated superior performance in controlling subvisible particles (2–300 μm), with all four formulations maintaining the lowest particle counts among tested groups despite showing expected increases under stress conditions (Figure 10). This confirms trehalose’s effectiveness in preventing particle formation during FD and subsequent storage. Surfactant-enhanced formulation (I4) exhibited additional stabilization benefits when supplemented with 0.02% (*w/v*) polysorbate 20, showing significantly better inhibition of insoluble particle formation at 50 °C compared to trehalose-only formulation (I8). This suggested polysorbate 20 may interfere with intermolecular interactions between insulin molecules that lead to particle generation. Excipient-free controls (I1, I2 for insulin; G1 for glucagon) consistently generated higher particle loads post-FD (Figure 10 and Figure 11), underscoring the necessity of lyoprotectants in preventing subvisible particle formation during FD processes. Arginine-containing glucagon formulations (G6, G7) displayed compromised performance, with immediate post-FD formation of abundant 25–300 μm particles, indicating potential incompatibility between arginine and polypeptide stability during processing.

## 4. Discussion

As is well-established for freeze-dried mAbs, residual moisture content is equally crucial for freeze-dried polypeptides. Formulations lacking excipients or lyoprotectants (G1, I1, I2) exhibited residual moisture levels exceeding 20%, indicating that the absence of structural excipients prevented the formation of a robust mico-structure during drying, thereby leading to cake collapse. This structural failure impeded the normal sublimation of ice crystals, particularly those located at the bottom of the cake, resulting in significantly elevated moisture content compared to other formulations. The abnormally high moisture levels (G1, I1 and I2) likely contributed to chemical instability, such as hydrolysis or oxidation, during accelerated stability studies. Consequently, these formulations demonstrated a sharp decline in the purity of both glucagon and insulin, along with a substantial increase in subvisible particle counts.

For freeze-dried mAb formulations, non-reducing disaccharides such as sucrose and trehalose form amorphous glass matrices that are critical for maintaining the chemical and physical stability of high-molecule-weight proteins. These matrices effectively protect proteins from denaturation, aggregation, and degradation during high-temperature processing and long-term storage. Inspired by these mAb formulation strategies, we systematically evaluated the impact of various formulation factors on the stability of polypeptides. Results demonstrated that, for insulin, both RP-HPLC and SE-HPLC analyses revealed reduced purity and increased the formation of aggregates and fragments in formulations without trehalose (I1, I2). In contrast, trehalose-containing formulations (I4, I6, I7, I8) effectively preserved the physical and chemical stability of insulin. Thermodynamically, this can be attributed to the high T_g_ of the remaining amorphous trehalose fraction, which elevates the overall T_g_ of the formulation. Kinetically, the amorphous trehalose serves as a glassy matrix, effectively restricting molecular mobility of insulin and thereby minimizing protein–protein interactions during storage. In contrast, HES, despite having highest T_g_ and remaining amorphous, failed to confer comparable stabilization. This is likely due to its inability to form compensatory hydrogen bonds with insulin or glucagon molecules after water removal, resulting in poor long-term stability of the HES-containing formulation (I5). XRPD results showed arginine remained amorphous state in the freeze-dried product. The effect of arginine on proteins stability appears to vary depending on the protein studied. Most previous studies reported that arginine suppresses lysozyme aggregation at neutral pH [54,55,56]. Moreover, arginine has been reported to exhibit a concentration-dependent mechanism, with varying effects on the stability of different proteins, indicating a relatively complex mode of action. At concentrations below 100 mM, arginine behaves similarly to glycine—stabilizing BSA, destabilizing myoglobin, and having a neutral effect on lysozyme. At higher concentrations, it induces nonspecific destabilization of all three proteins, analogous to but weaker than guanidinium ions [57]. Although fibrillation assays, DLS, and MFI demonstrated that arginine suppresses glucagon aggregation, it performed unsatisfactorily in RP-HPLC, with poor outcomes in key chemical stability indicators such as purity and recovery. As reported in the literature, the high concentration of arginine hydrochloride in this study (pH 3.0) led to a lower T_g_ (46.9 °C) [58], which thermodynamically explains the compromised stabilization of freeze-dried glucagon in arginine-containing formulations during accelerated stability testing at 40 °C and 50 °C. Notably, formulations containing 40 mg/mL arginine hydrochloride showed marked cake collapse after three months of accelerated stability studies, further accounting for the poor chemical stability of glucagon.

Although mannitol is a widely used excipient in low-molecular-weight pharmaceutical drugs, its inclusion in freeze-dried glucagon and insulin formulations significantly compromised the stability of both polypeptides. XRPD patterns revealed that the presence of MHH signals in I3 formulation, which may release moisture during storage and thereby increase the chemical instability of insulin, leading to a decrease in purity. However, given the relatively low intensity of the MHH signals, it is inferred that this is not the primary cause of insulin instability. DSC results indicated that no detectable T_g_ in formulations containing mannitol (G2 and I3), suggesting complete crystallization of mannitol in these systems. Extensive mannitol crystallization may disturb the higher-order structures of insulin and glucagon due to ice–water interface effects during the freezing process [59,60]. Furthermore, accelerated stability studies revealed that mannitol-containing formulations (G2 and I3) exhibited a significant decline in polypeptide purity and a notable increase in subvisible particles. This is likely due to the inability of the crystalline mannitol matrix to form an amorphous stabilizing scaffold, unlike trehalose, which acts as an effective lyoprotectant by minimizing polypeptide–polypeptide interactions. It has been reported that as the mass ratio of amorphous excipient sucrose to crystalline excipient mannitol increases in a protein formulation, the degree of crystallinity of mannitol decreases significantly, indicating that sucrose can substantially suppress mannitol crystallization [23]. The same study also pointed out that the inclusion of an annealing step could promote the crystallization of mannitol, thereby compromising the long-term stability of the protein [61]. Therefore, a rational formulation strategy could involve combining amorphous and crystalline excipients while omitting the annealing step during FD, which is expected to enhance drying efficiency while maximizing the long-term stability of the freeze-dried polypeptide.

In summary, despite the difference of approximately 30 amino acids and distinct higher-order structures between insulin and glucagon, the fundamental mechanisms through which formulation components—such as amorphous and crystalline excipients, as well as surfactants—exert their stabilizing or destabilizing effects are consistent with those observed for high-molecular-weight mAbs. Trehalose—a widely adopted excipient in freeze-dried mAb formulations—proves effective in stabilizing polypeptides by forming amorphous matrix that decrease the interaction of polypeptides and maintain native conformation. On the other hand, crystalline excipients such as mannitol, which undergo crystallization during processing and storage, exhibit a reduced capacity to engage in stabilizing interactions with polypeptides, thereby offering limited protection against degradation. Consequently, a rational formulation strategy involves the combination of crystalline and amorphous excipients in specific ratios to optimize the stability of freeze-dried polypeptide formulations. These findings underscore the importance of excipient selection based on molecular interaction potential, rather than thermal properties alone, and provide a rational framework for designing stable freeze-dried polypeptide therapeutics.

## 5. Conclusions

This study systematically evaluated the effects of various excipients on the stability of freeze-dried polypeptide formulations using insulin and glucagon as model drugs. Non-reducing disaccharides, particularly trehalose, were identified as essential for maintaining both physical and chemical integrity. The use of mannitol as a sole bulking agent proved insufficient to form a stable amorphous matrix during storage, failing to suppress polypeptide-polypeptide interactions and resulting in poor long-term stability. The results further establish the superior stabilizing effect of trehalose, which forms an amorphous glassy matrix that restricts molecular mobility, thereby inhibiting protein denaturation, aggregation, and chemical degradation. Polysorbate 20 also contributed to polypeptides stability by significantly reducing the formation of subvisible particles. An optimal formulation strategy involves the combination of amorphous and crystalline excipients at an optimized mass ratio. This approach leverages the capacity of crystalline agents to enhance FD efficiency and produce pharmaceutically elegant cakes, while simultaneously utilizing amorphous protectants to ensure the long-term stability of the polypeptides.

These findings provide a solid theoretical foundation and practical guidance for excipient selection in the FD of polypeptide-based pharmaceuticals. Future studies should focus on exploring synergistic effects among excipients and further elucidating their molecular protection mechanisms to enable rational formulation design and facilitate commercial production.

## Figures and Tables

**Table 1:** The overview of FD formulations.

Protein	No.	Formulation					
		Polysorbate 20	Mannitol (mg/mL)	Trehalose (mg/mL)	Hydroxyethyl Starch (mg/mL)	Sorbitol (mg/mL)	Arginine Hydrochloride (mg/mL)
Glucagon	G1	0.02%	—	—	—	—	—
	G2	0.02%	40	—	—	—	—
	G3	0.02%	—	40	—	—	—
	G4	0.02%	—	—	40	—	—
	G5	0.02%	—	40	—	2.0	—
	G6	0.02%	—	40	—	—	2.4
	G7	0.02%	—	—	—	—	40
Insulin	I1	—	—	—	—	—	—
	I2	0.02%	—	—	—	—	—
	I3	0.02%	40	—	—	—	—
	I4	0.02%	—	40	—	—	—
	I5	0.02%	—	—	40	—	—
	I6	0.02%	—	40	—	2.0	—
	I7	0.02%	—	40	—	—	2.4
	I8	—	—	40	—	—	—

**Table 2:** The overview of FD cycle.

No.	Stage	Shelf Temperature (°C)	Ramp Rate (°C/min)	Hold Time (min)	Chamber Pressure (mTorr)
1	Freezing	−45	0.56	60	—
2	Annealing	−15	0.86	120	—
3	Freezing	−45	1.00	75	—
4	Primary drying	−25	0.16	1400	60
5	Secondary drying	30	0.22	300	60

**Table 3:** Elution gradient of mobile phase for RP-HPLC.

Glucagon			Insulin		
Time (min)	A (%)	B (%)	Time (min)	A (%)	B (%)
0.0	70.0	30.0	0.0	71.0	29.0
30.0	64.0	36.0	30.0	63.0	37.0
30.1	10.0	90.0	35.0	35.0	65.0
31.0	10.0	90.0	35.1	71.0	29.0
31.1	70.0	30.0	48.0	71.0	29.0
45.0	70.0	30.0			

**Table 4:** The residual moisture of freeze-dried samples of different samples (n = 3).

Formulation	Moisture (%)	Formulation	Moisture (%)
G1	19.27 ± 8.20	I1	28.03 ± 1.82
G2	2.66 ± 0.88	I2	25.86 ± 4.53
G3	1.78 ± 0.85	I3	1.89 ± 0.48
G4	1.37 ± 0.64	I4	2.49 ± 0.22
G5	1.52 ± 0.49	I5	1.54 ± 0.17
G6	1.53 ± 0.30	I6	2.17 ± 0.39
G7	2.05 ± 0.41	I7	2.01 ± 0.11
		I8	1.96 ± 0.06

**Table 5:** The T_g_ of freeze-dried samples of different samples (n = 3).

Formulation	T_g_ (°C)	Formulation	T_g_ (°C)
G1	/	I1	/
G2	/	I2	/
G3	72.7 ± 0.3	I3	/
G4	200.6 ± 1.7	I4	68.8 ± 8.5
G5	63.7 ± 2.4	I5	205.8 ± 1.8
G6	69.1 ± 0.4	I6	57.6 ± 4.9
G7	46.9 ± 1.1	I7	61.2 ± 4.3
		I8	61.2 ± 2.9

**Table 6:** The T_g_′ of freeze-dried samples of different samples (n = 3).

Formulation	T_g_′ (°C)	Formulation	T_g_′ (°C)
G1	/	I1	/
G2	−34.5 ± 0.1	I2	/
G3	−30.1 ± 0.2	I3	−31.7 ± 0.2
G4	−12.1 ± 0.0	I4	−33.2 ± 0.0
G5	−43.9 ± 0.0	I5	−15.4 ± 0.0
G6	−31.1 ± 0.0	I6	−33.6 ± 0.0
G7	/	I7	−32.9 ± 0.2
		I8	−32.4 ± 0.0

## Data Availability

The raw data supporting the conclusions of this article will be made available by the authors on request.

## Abbreviations

AA, amino acid; DLS, dynamic lighting scattering; DSC, differential scanning calorimetry; FD, freeze-drying; HES, hydroxyethyl starch; mAb, monoclonal antibody; MFI, micro-flow imaging; RP-HPLC, reversed phase high-performance liquid chromatography; SD, standard deviation; SE-HPLC, size exclusion high-performance liquid chromatography; SMD, small molecule drug; T_g_′, the glass transition temperatures of the frozen solutions; T_g_, the glass transition temperatures of the freeze-dried solids; ThT, Thioflavin T; XRPD, X-ray powder diffraction.
